# Supplementary material for: Effect of enterally administered sleep-promoting medication on the intravenous sedative dose and its safety and cost profile in mechanically ventilated patients: A retrospective cohort study
Source: PLoS One. 2021 Dec 20;16(12):e0261305. doi: 10.1371/journal.pone.0261305 (PMC8687529; doi:10.1371/journal.pone.0261305)
Supplement: S2 File — (DOCX) [file pone.0261305.s002.docx]

| Supporting Table 2. Univariate analysis of study outcomes | | | | | | |
| --- | --- | --- | --- | --- | --- | --- |
|  | EA group vs. LA group |  | EA group vs. NA group |  | LA group vs. NA group |  |
|  | differences (95% CI) | p | differences (95% CI) | p | differences (95% CI) | p |
| Primary outcome |  |  |  |  |  |  |
| Average daily propofol dose,   mg/kg/day | β, -5.25 (-9.18 to -1.32) | <0.01 | β, -4.84 (-9.09 to -0.60) | 0.03 | β, -0.40 (-4.37 to 3.56) | 0.84 |
| Secondary outcome |  |  |  |  |  |  |
| MV through an ET tube, days | β, -1.21 (-3.08 to 0.67) | 0.20 | β, -0.39 (-2.41 to 1.64) | 0.71 | β, 0.82 (-1.06 to 2.71) | 0.39 |
| Length of ICU stay, days | β, -2.06 (-5.77 to 1.66) | 0.28 | β, -3.35 (-7.37 to 0.65) | 0.10 | β, -1.30 (-5.05 to 2.44) | 0.49 |
| RASS ≧ 2 points | OR, 0.63 (0.27 to 1.49) | 0.29 | OR, 0.68 (0.27 to 1.72) | 0.42 | OR, 1.08 (0.46 to 2.55) | 0.86 |
| Delirium | OR, 0.71 (0.28 to 1.83) | 0.49 | OR, 0.84 (0.31 to 2.32) | 0.74 | OR, 1.17 (0.47 to 2.94) | 0.74 |
| SPM, sleep-promoting medication; MV, mechanical ventilation; CI, confidence interval; IQR, interquartile range; β, β coefficient; OR, odds ratio; ET, endotracheal; RASS, Richmond Agitation-Sedation Scale; IV, intravenous | | | | | | |
