# Supplementary material for: Effect of enterally administered sleep-promoting medication on the intravenous sedative dose and its safety and cost profile in mechanically ventilated patients: A retrospective cohort study
Source: PLoS One. 2021 Dec 20;16(12):e0261305. doi: 10.1371/journal.pone.0261305 (PMC8687529; doi:10.1371/journal.pone.0261305)
Supplement: S3 File — (DOCX) [file pone.0261305.s003.docx]

| Supporting Table 3. Comparison between all patients receiving and not receiving SPM | | | | |
| --- | --- | --- | --- | --- |
|  | SPM group | No SPM group | SPM group vs. no SPM group | p value |
|  | (n=87) | (n=36) | differences (95% CI) |  |
| Primary outcome^a^ |  |  |  |  |
| Average daily propofol dose,   median [IQR], mg/kg/day | 3.1 [1.6 to 11.0] | 6.9 [2.7 to 11.7 ] | β, -1.58 (-5.14 to 1.97) | 0.38 |
| Secondary outcome^b^ |  |  |  |  |
| MV duration through an oral ET  tube, median [IQR], days | 6 [5 to 9] | 6 [4 to 8] | β, 0.79 (-0.92 to 2.49) | 0.36 |
| Length of ICU stay,   median [IQR], days | 9 [7 to 13] | 9 [6 to 11] | β, -1.20 (-4.38 to 1.98) | 0.46 |
| RASS 2 points and more, n (%) | 41 (47) | 18 (50) | OR, 1.05 (0.44 to 2.50) | 0.91 |
| delirium, n (%) | 27 (31) | 11 (31) | OR, 1.26 (0.48 to 3.32) | 0.64 |
| SPM, sleep-promoting medication; Cl, confidence interval; IQR, interquartile range; β, β coefficient; MV, mechanical ventilation; ET, endotracheal; ICU, intensive care unit; RASS, Richmond Agitation-Sedation Scale; OR, odds ratio; ICU, intensive care unit; SOFA, sequential organ failure assessment; IV, intravenous; EN, enteral  a Multivariate analysis adjusted for age, sex, Charlson comorbidity index, pre-admission use of sleeping pills, diagnosis on ICU admission, maximum SOFA scores during the first week after admission, IV dexmedetomidine administration, IV midazolam administration, EN ramelteon administration, EN benzodiazepines administration, average daily fentanyl dose per body weight, and acetaminophen administration. b Multivariate analysis adjusted for age, sex, Charlson comorbidity index, maximum SOFA scores during the first week after admission, diagnosis on ICU admission, and IV midazolam administration. | | | | |
