# Supplementary material for: Effect of enterally administered sleep-promoting medication on the intravenous sedative dose and its safety and cost profile in mechanically ventilated patients: A retrospective cohort study
Source: PLoS One. 2021 Dec 20;16(12):e0261305. doi: 10.1371/journal.pone.0261305 (PMC8687529; doi:10.1371/journal.pone.0261305)

Supporting File 4. Normal Q-Q plot

normal Q-Q plot for primary outcome

<The average daily propofol dose per body weight >


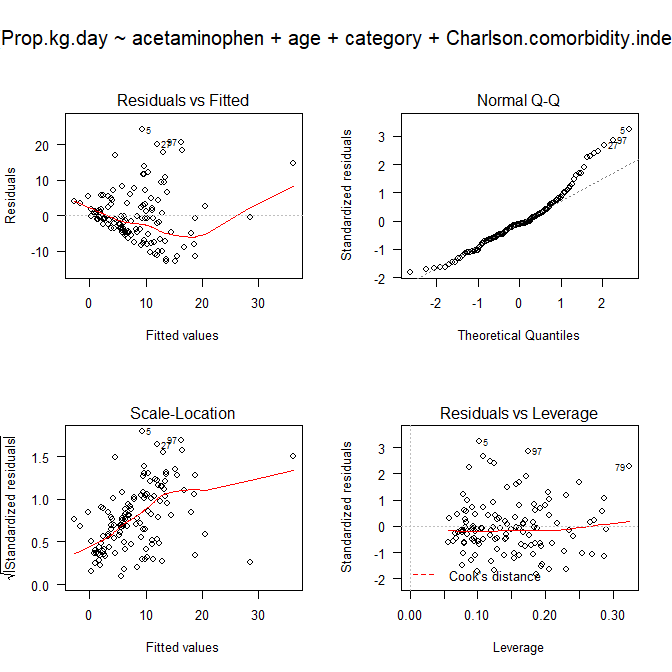


normal Q-Q plot for secondary outcome

<Mechanical ventilation duration>


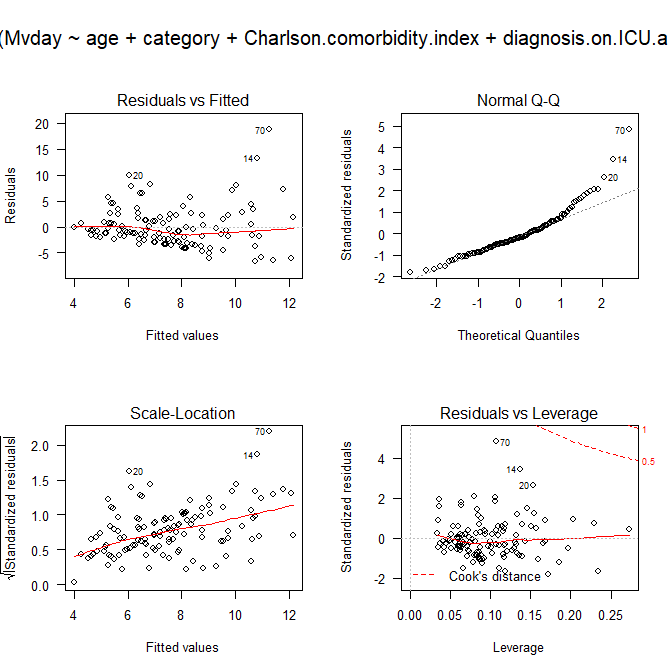


<Length of ICU stay>


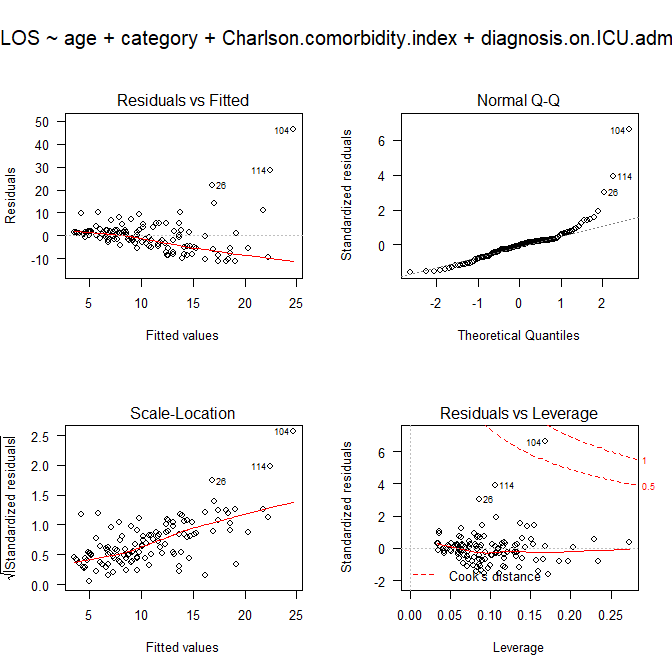

Supplement: S4 File — (DOCX) [file pone.0261305.s004.docx]
