# Supplementary material for: Effect of enterally administered sleep-promoting medication on the intravenous sedative dose and its safety and cost profile in mechanically ventilated patients: A retrospective cohort study
Source: PLoS One. 2021 Dec 20;16(12):e0261305. doi: 10.1371/journal.pone.0261305 (PMC8687529; doi:10.1371/journal.pone.0261305)
Supplement: S6 File — (DOCX) [file pone.0261305.s006.docx]

研究課題名

「人工呼吸器管理中の患者における睡眠薬の経腸投与と鎮静薬の経静脈投与量の変化及び予後に関する後方視的コホート研究」

研究責任者所属：救急・集中治療科

研究責任者名：恒光　健史

Ver.1.0 　2020年5月25日

目次

[1. 研究の目的 2](#_Toc421613394)

[2. 研究の背景及び意義 2](#_Toc421613395)

[3. 研究対象者及び適格性の基準 2](#_Toc421613396)

[4. 研究の方法 2](#_Toc421613397)

[5. 評価項目](#_Toc421613398) 2

[6. 統計的事項 3](#_Toc421613399)

[7. スケジュールあるいは研究期間 3](#_Toc421613400)

[8. 研究対象者に生じる負担並びに予測されるリスク及び利益 3](#_Toc421613401)

[9. 研究の変更、中止・中断、終了 3](#_Toc421613402)

[10. 研究の科学的合理性の根拠](#_Toc421613403) 3

[11. 研究対象者からインフォームド・コンセントを受ける手続き 3](#_Toc421613404)

[12. 個人情報等の取扱い 3](#_Toc421613405)

[13. 情報の保管及び廃棄の方法 4](#_Toc421613406)

[14. 院長への報告内容及び方法 4](#_Toc421613407)

[15. 研究対象者の費用負担、謝礼 4](#_Toc421613408)

[16. 研究結果の公表 4](#_Toc421613409)

[17. 研究資金及び利益相反 4](#_Toc421613410)

[18. 研究対象者等及びその関係者からの相談等への対応 4](#_Toc421613411)

[19. 研究業務の委託 4](#_Toc421613412)

[20. 研究の実施体制 4](#_Toc421613413)

[21. 参考資料・文献リスト 5](#_Toc421613414)

# **研究の目的**

　気管挿管され人工呼吸管理が実施された患者の睡眠薬の経腸投与と鎮静薬の経静脈投与量の変化及び予後の関係を検討する。

# **研究の背景及び意義**

睡眠障害は、ICUに入院した重症患者でしばしば観察される[1-3]が、患者に悪影響を及ぼし、予後を悪化させる可能性がある。睡眠障害は、重症患者において重要な役割を果たす免疫および代謝性内分泌機能の異常を引き起こす [4,5]。さらに、せん妄の発生[6]、非侵襲的人工呼吸不全[7]、および死亡率の増加[8]と関連している可能性がある。その結果、最近の臨床ガイドラインでは、睡眠を改善するための研究方法の必要性が示されている[9]。

プロポフォールやベンゾジアゼピンなどの静脈内鎮静剤は、睡眠効率を改善するために投与されるが[10]、重症患者に悪影響を及ぼす可能性がある。静脈内鎮静薬は、無呼吸および低酸素を引き起こす呼吸機能および徐脈および低血圧を引き起こす循環機能に影響を与える [11-13]。さらに、鎮静剤は機械的換気の延長に関連している可能性がある [14]。さらに、静脈内鎮静薬は深い鎮静を引き起こす傾向があり [15] 、これは予後の悪化と関連している [16,17]。

メラトニン、ラメルテオン、非定型抗精神病薬を含む睡眠促進薬（SPM）は、重症患者の睡眠効率を改善する可能性がある [9]。パイロットランダム化比較試験では、メラトニンが夜間の睡眠効率を改善する可能性があることが報告されている[18]。安全性に関しては、いくつかの研究で安全な経腸投与が可能である報告されている[19,20]。SPMの経腸投与は、重症患者に対して安全に実施できる可能性がある。

現在のところ、SPM経腸投与と静脈内鎮静剤の量との関係、SPM経腸投与の有用性と安全性に関する研究はない。我々は，SPM経腸投与が睡眠を促進し，睡眠のために投与される静脈内鎮静剤量を減少させるという仮説を立てた。本探索的研究では、SPM経腸投与と睡眠時の静脈内鎮静剤投与量との関係を検討した。さらに、SPM経腸投与の安全性とコストを検討することを目的とした。本研究は、機械換気患者に対するSPM経腸投与の効果を理解する上で重要な研究であると考えている。

# **研究対象者及び適格性の基準**

1. セッティング

施設：兵庫県立尼崎総合医療センター

期間：2015年7月から2020年1月

2）適格基準

選択基準(以下のすべてを満たす)

・気管挿管と48時間以上の人工呼吸管理の実施

・ICU入室後24時間以内に人工呼吸器管理を開始

・15歳以上

除外基準（以下のいずれかを満たす）（DPC病名および必要に応じてカルテレビューを行う）

・脳卒中（脳出血、脳梗塞、くも膜下出血）

・意識障害を伴う頭部外傷

・中枢神経疾患（てんかん、髄膜炎など）

・心肺停止

・薬物中毒

・腸管が使用不可（消化管の手術、イレウス、消化管出血、持続的な筋弛緩薬を使用）

・精神疾患や認知症

・child C 肝硬変

・妊娠

・ICU再入室

・体重が未測定

・もともと気管切開術が実施されている

・ICUを死亡退室

・鎮静薬としてプロポフォールを使用していない

3) サンプルサイズ（対象者の人数）およびその算定根拠

120例程度、研究期間内の当院における症例の数から目標症例数を決定する。

# **研究の方法**

4.1.研究の種類・デザイン

後方視的コホート研究

4.2.研究・調査項目

主要な要因の定義、測定方法

＜exposure＞

睡眠薬の経腸投与（トラゾドン、テトラミド、クエチアピン、ベルソムラ[21-23]）

（実施時刻が夕食後以降であれば睡眠改善目的の投与と考える）

「投与が入室後48時間以内」と「投与が入室後48時間以降」と「投与なし」の3群に分ける。

48時間で区切った理由としては睡眠障害の病態生理を考慮すると早期の睡眠管理がより有益であるように思われること[24]、経腸栄養開始時に経腸薬を投与できること[25,26]などから、カットオフを48時間とした。SPMは主治医の判断で投与した。本研究では、ラメルテオンとベンゾジアゼピン系薬剤は曝露として考慮しなかった。これは、ラメルテオンの短期投与で有意な睡眠改善が認められたものの、その差は小さかった[27]ためである。さらに、ラメルテオンに類似した薬剤であるメラトニンは、睡眠の質の改善に関するエビデンスが不十分であり[28]、欧州のガイドラインでは強く推奨されていない[22]ためである。ICUでのベンゾジアゼピン系薬剤の使用は有害事象の発生率が高い[29]ため睡眠薬から除外した。その他の抗精神病薬（ハロペリドール、リスペリドンなど）は、主に動揺のために投与され、我々のICUでは睡眠改善のために使用されることはほとんどなかったため、曝露から除外した。

＜primary outcome＞

経静脈的な持続鎮静薬（プロポフォール）の一日の体重あたりの平均使用量

（求め方：各患者の人工呼吸器管理期間に使用した量を体重と人工呼吸器管理されていた日数で割った値）

プロポフォールを選択したのは、プロポフォールがICUで最も一般的に使用されている鎮静剤であり、世界的に使用量が増加しているためである [30]。継続的な鎮静剤は1時間以上投与されるものと定義した。当直の医師が鎮静薬投与量の目標Richmond Agitation-Sedation Scale（RASS）を設定し、看護師が主にイニシアチブをとって増減させた。

＜other outcome＞

・経口気管チューブを用いた機械換気の持続時間

・ICU滞在期間

・ICUの混乱評価法または集中ケアせん妄スクリーニングチェックリストに基づいて診断されたせん妄

・経口気管チューブを用いた機械換気中にRASS≧2点と定義された不穏

＜adverse enent＞

・自己抜管の有無

・人工呼吸器管理中にその他の経静脈的な鎮静薬（ハロペリドール、アタラックスP、サイレース）の投与の有無

（データ収集方法：megaoak ）

・薬剤変更を要する肝障害の有無

・心室性不整脈の有無

＜cost＞

費用は、2020年6月の薬価を基準に、経口気管内挿管による機械的人工呼吸中に本試験で抽出した経腸剤及び静脈内投与剤のそれぞれの量を合算して算出する。円からドルへの換算は、2020年6月時点のレートである1ドル108円で算出する。

その他、研究対象者について、下記の臨床情報を診療録より取得する。

鎮痛剤（アセトアミノフェン、フェンタニル）、その他の静脈内鎮静剤（ミダゾラム、デクスメデトミジン）、その他の神経活性経腸薬（ラメルテオン、ヨクカンサン、ペロスピロン、リスペリドン、ベンゾジアゼピン）の投与に関するデータを収集する。

＜交絡因子＞

年齢、性、入院前の睡眠薬の使用、ICU入院時の診断、入院後1週間の最大SOFAスコア、デクスメデトミジン静注、ミダゾラム静注、ラメルテオン経腸投与、ベンゾジアゼピン経腸投与、フェンタニル1日平均体重当たり投与量、およびアセトアミノフェン投与

# **統計的事項**

5.1. 統計解析の方法

連続データは中央値と四分位の範囲を用いて記述し、カテゴリカルデータは度数とパーセンテージを用いて記述した。我々は重回帰モデルを構築し、SPMの経腸投与と体重当たりの平均プロポフォール1日量との関連を検討した。主要研究アウトカムの共変量として以下の変数を設定した：年齢、性、入院前の睡眠薬の使用、ICU入院時の診断、入院後1週間の最大逐次臓器不全評価（SOFA）スコア、デクスメデトミジン静注、ミダゾラム静注、ラメルテオン経腸投与、ベンゾジアゼピン経腸投与、フェンタニル1日平均体重当たり投与量、およびアセトアミノフェン投与。その他のアウトカムの解析には、バイナリ変数にはロジスティック回帰分析を用い、連続変数には重回帰線形モデルを用いた。二次試験の共変量として、年齢、性別、Charlson併存疾患指数、入院後1週間のSOFAスコア、ICU入院時の診断、ミダゾラムの静脈内投与を設定した。すべての検定は両側検定で、有意水準は0.05であった。解析はEZR（バージョン：1.36）[31]を用いて行った。

# **スケジュールあるいは研究期間**

臨床研究審査委員会承認日～西暦2022年12月

（調査対象期間：西暦2015年7月～2020年1月）

# **遵守すべき倫理指針**

厚生労働省、文部科学省の「人を対象とする医学系研究に関する倫理指針」に従う。

# **研究対象者に生じる負担並びに予測されるリスク及び利益**

8.1. 負担並びに予測されるリスク

本研究に参加することによる研究対象者に生じる負担並びにリスクはない。

8.2. 予測される利益

本研究に参加することによる研究対象者個人への直接的な利益は生じない。研究の成果は、将来の人工呼吸器管理が必要な患者の良好な鎮静方法の進歩に有益となる可能性がある。

# **研究の変更、中止・中断、終了**

9.1. 研究の変更

研究実施計画書の変更または改訂を行う場合は、あらかじめ倫理委員会の承認を必要とする。

9.2. 研究の中止、中断

研究責任者は、倫理委員会により中止の勧告あるいは指示があった場合は、研究を中止する。また、研究の中止または中断を決定した時は、速やかに院長にその理由とともに文書で報告する。

9.3. 研究の終了

研究の終了時には、研究責任者は速やかに研究終了報告書を院長に提出する。

# **研究対象者からインフォームド・コンセントを受ける手続き**

本研究は、新たに試料・情報を取得することはなく、既存情報のみを用いて実施する研究であるため、研究対象者から文書または口頭による同意は得ない。研究についての情報を研究対象者に公開（病院内に掲示）し、研究が実施されることについて、研究対象者が拒否できる機会を保障する。

# **個人情報等の取扱い**

研究に携わる者は、個人情報の取扱いに関して、「人を対象とする医学系研究に関する倫理指針」、「個人情報の保護に関する法律」及び適用される法令、条例等を遵守する。調査により得られた情報を取扱う際は、研究対象者の秘密保護に十分配慮し、特定の個人を識別することができないよう、研究対象者に符号もしくは番号を付与する。対応表は研究責任者が電子カルテネットワーク上に保管し、ネットワーク外に個人を識別することができる情報の持ち出しは行わない。解析に際しては、匿名加工情報のみを個人のコンピューターを用いて解析する。拒否があった場合には、研究責任者が匿名加工情報からの削除を行なう。

本研究結果が公表される場合にも、研究対象者個人を特定できる情報を含まないこととする。

また、本研究の目的以外に、本研究で得られた情報を利用しない。

# **情報の保管及び廃棄の方法**

【情報の管理】

研究責任者は、研究等の実施に関わる文書（申請書類の控え、通知文書、研究対象者識別コードリスト、同意書、症例報告書、その他データの信頼性を保証するのに必要な書類または記録など）を医局の鍵のかかるロッカーに保管する。

保管期間は、研究の終了について報告された日から5年を経過した日又は研究結果の最終の公表について報告された日から3年を経過した日のいずれか遅い日までの期間とする。

保管期間終了後に紙媒体に関してはシュレッダーで裁断し破棄する。その他媒体に関しては適切な方法で破棄する。

# **院長への報告内容及び方法**

以下の場合に文書にて院長に報告する。

1. 研究の倫理的妥当性若しくは科学的合理性を損なう事実若しくは情報又は損なうおそれのある情報であって研究の継続に影響を与えると考えられるものを得た場合
2. 研究の実施の適正性若しくは研究結果の信頼を損なう事実若しくは情報又は損なうおそれのある情報を得た場合
3. 研究を終了（中止）した場合
4. 研究の進捗状況（年に1回）

# **研究対象者の費用負担、謝礼**

研究に参加することによる研究対象者の費用負担は発生しない。また、謝礼も発生しない。

# **研究結果の公表**

国際・国内学会および論文発表を行う。公表時には、個人を識別する情報は一切使用しないように十分に配慮する。

# **研究資金及び利益相反**

本研究は、研究責任者が所属する診療科の研究資金で実施する。また、本研究の研究者は、「県立尼崎総合医療センター利益相反管理規定」に従って、臨床研究利益相反委員会に必要事項を申告し、その審査と承認を得るものとする。

# **研究対象者等及びその関係者からの相談等への対応**

研究対象者やその関係者からの相談窓口は、研究責任者とし、連絡先を公開文書に記載する。

# **研究業務の委託**

研究に関する業務の委託はない。

# **研究の実施体制**

兵庫県立尼崎総合医療センター 救急集中治療科

研究責任者　救急科　医長　恒光健史

　　 呼吸器内科　医長　片岡裕貴

# **参考資料・文献リスト**

1. Cooper AB, Thornley KS, Bryan Young G, et al. Sleep in Critically Ill Patients Requiring Mechanical Ventilation. Chest. 2000;117(3):809-18.
2. Freedman NS, Gazendam J, Levan L, et al. Abnormal Sleep/Wake Cycles and the Effect of Environmental Noise on Sleep Disruption in the Intensive Care Unit. Am J Respir Crit Care Med. 2001;163(2):451-7.
3. Elliott R, McKinley S, Cistulli P, et al. Characterisation of sleep in intensive care using 24-hour polysomnography: An observational study. Crit Care. 2013;17(2):R46.
4. Spiegel K, Leproult R, Cauter E Van. Impact of sleep debt on metabolic and endocrine function. Lancet. 1999;354(9188):1435-9.
5. Spiegel K, Sheridan JF, Van Cauter E. Effect of sleep deprivation on response to immunization. JAMA. 2002;288:1471–2.
6. Fadayomi AB, Ibala R, Bilotta F, et al. A Systematic Review and Meta-Analysis Examining the Impact of Sleep Disturbance on Postoperative Delirium. Crit Care Med. 2018;46(12):e1204-e1212.
7. Campo FR, Drouot X, Thille AW, et al. Poor sleep quality is associated with late noninvasive ventilation failure in patients with acute hypercapnic respiratory failure. Crit Care Med. 2010;38(2):477-85.
8. Boyko Y, Toft P, Ørding H, et al. Atypical sleep in critically ill patients on mechanical ventilation is associated with increased mortality. Sleep Breath. 2019;23:379–88.
9. Devlin JW, Skrobik Y, Gélinas C, et al. Clinical Practice Guidelines for the Prevention and Management of Pain, Agitation/Sedation, Delirium, Immobility, and Sleep Disruption in Adult Patients in the ICU. Crit Care Med. 2018;46(9):e825-e873.
10. Brito RA, do Nascimento Rebouças Viana SM, Beltrão BA, et al. Pharmacological and non-pharmacological interventions to promote sleep in intensive care units: a critical review. Sleep Breath. 2020;24(1):25-35.
11. McCollum JS, Dundee JW, Halliday NJ, et al. Dose response studies with propofol ('Diprivan’) in unpremedicated patients. Postgrad Med J. 1985;61 Suppl 3:85-7.
12. Xia ZQ, Chen SQ, Yao X, et al. Clinical benefits of dexmedetomidine versus propofol in adult intensive care unit patients: A meta-analysis of randomized clinical trials. J Surg Res. 2013;185(2):833-43.
13. Chawla N, Boateng A, Deshpande R. Procedural sedation in the ICU and emergency department. Curr Opin Anaesthesiol. 2017;30(4):507-512.
14. Kollef MH, Levy NT, Ahrens TS, et al. The use of continuous IV sedation is associated with prolongation of mechanical ventilation. Chest. 1998;114:541–8.
15. Jackson DL, Proudfoot CW, Cann KF, et al. The incidence of sub-optimal sedation in the ICU: A systematic review. Crit Care. 2009;13(6):R204.
16. Shehabi Y, Bellomo R, Kadiman S, et al. Sedation intensity in the first 48 hours of mechanical ventilation and 180-day mortality: A multinational prospective longitudinal cohort study. Crit Care Med. 2018;46(6):850-859.
17. Tanaka LMS, Azevedo LCP, Park M, Schettino G, et al. Early sedation and clinical outcomes of mechanically ventilated patients: A prospective multicenter cohort study. Crit Care. 2014;18(4):R156.
18. Bourne RS, Mills GH, Minelli C. Melatonin therapy to improve nocturnal sleep in critically ill patients: Encouraging results from a small randomised controlled trial. Crit Care. 2008;12(2):R52.
19. Dube KM, DeGrado J, Hohlfelder B, et al. Evaluation of the Effects of Quetiapine on QTc Prolongation in Critically Ill Patients. J Pharm Pract. 2018;31(3):292-297.
20. Hatta K, Kishi Y, Wada K, et al. Preventive effects of suvorexant on delirium: A randomized placebo-controlled trial. J Clin Psychiatry. 2017;78(8):e970-e979.
21. Yi X yan, Ni S fen, Ghadami MR, et al. Trazodone for the treatment of insomnia: a meta-analysis of randomized placebo-controlled trials. Sleep Med. 2018;45:25-32.
22. Riemann D, Baglioni C, Bassetti C, et al. European guideline for the diagnosis and treatment of insomnia. J Sleep Res. 2017;26:675–700.
23. Herring WJ, Connor KM, Snyder E, et al. Effects of suvorexant on the Insomnia Severity Index in patients with insomnia: analysis of pooled phase 3 data. Sleep Med. 2019;56:219-223.
24. Pisani MA, D’Ambrosio C. Sleep and Delirium in Critically Ill Adults: A Contemporary Review. Chest. 2020;157(4):977-984.
25. McClave SA, Taylor BE, Martindale RG, et al. Guidelines for the Provision and Assessment of Nutrition Support Therapy in the Adult Critically Ill Patient: Society of Critical Care Medicine (SCCM) and American Society for Parenteral and Enteral Nutrition (A.S.P.E.N.). J Parenter Enter Nutr. 2016;40:159–211.
26. Singer P, Blaser AR, Berger MM, et al. ESPEN guideline on clinical nutrition in the intensive care unit. Clin Nutr. 2019;38(1):48-79.
27. Kuriyama A, Honda M, Hayashino Y. Ramelteon for the treatment of insomnia in adults: A systematic review and meta-analysis. Sleep Med. 2014;15(4):385-92.
28. Lewis SR, Pritchard MW, Schofield-Robinson OJ, et al. Melatonin for the promotion of sleep in adults in the intensive care unit. Cochrane Database Syst Rev. 2018;5(5):CD012455.
29. Kok L, Slooter AJ, Hillegers MH, et al. Benzodiazepine use and neuropsychiatric outcomes in the ICU: A systematic review. Crit. Care Med. 2018;46(10):1673-1680.
30. Owen GD, Stollings JL, Rakhit S, et al. International analgesia, sedation, and delirium practices: A prospective cohort study. J Intensive Care. 2019;7:25.
31. Kanda Y. Investigation of the freely available easy-to-use software “EZR” for medical statistics. Bone Marrow Transplant. 2013;48:452–8.

臨床研究に関する公開情報

令和２年５月25日

疫学研究とは、病気にかかることの頻度や病気の多さを調べて、その原因を明らかにする研究です。私たちは過去のカルテより得られた情報を利用して、現在まで行われた病気の診断・治療の評価を行い、より良い診断・治療法を確立し患者さんに還元できるように、下記の疫学研究を行っています。

下記の疫学研究は、兵庫県立尼崎総合医療センター倫理委員会の承認を得た後、研究責任者の管轄のもとに行われます。当院にすでに記録されている臨床情報をもとに行われるため、対象となる患者さんに新たにご負担をおかけすることはありません。

また、この研究の結果は専門の学会や学術雑誌に発表されることがありますが､対象者のプライバシーは十分に尊重され、個人に関する情報(氏名など)が外部に公表されることは一切ありません。

もし，下記の疫学研究にご自身の臨床情報を使用されることに同意されない方は，下記連絡先にご連絡くだされば，解析対象から除外させていただきます。同意されない場合でも、診療上であなたが不利益を被ることは一切ありません。また下記研究に関して、ご不明な点がございましたら、いつでも下記連絡先にお問い合わせください。

＜概要＞

研究課題名:

「人工呼吸器管理中の患者における睡眠薬の経腸投与と鎮静薬の経静脈投与量の変化及び予後に関する後方視的コホート研究」

研究期間：2015年7月から2022年3月まで

対象：2015年7月から2019年１２月に兵庫県立尼崎総合医療センターに入院した中枢神経障害患者

研究目的： 気管挿管され人工呼吸管理が実施された患者の睡眠薬の経腸投与時期と鎮静薬の経静脈投与量の変化及び予後の関係を検討します。

方法：診療記録より臨床情報を収集します。収集する臨床情報には、年齢、性別、BMI、来院時のGCS、慢性疾患の有無（呼吸不全、心不全、透析、癌や免疫抑制、肝硬変）、ICU入室時の各SOFAスコア（循環、呼吸、肝、腎、凝固、意識）、SOFAスコアの合計、疾患名、院内死亡、ICU死亡、ICU滞在期間、人工呼吸器期間、入院期間、気管切開、鎮静深度（RASS）、不整脈、使用した薬剤の情報など

研究成果は学会、および論文にて公表します。

個人情報：臨床情報は匿名化され、個人が特定できないようにして、必要な臨床データのみを収集して解析を行います。そのため、本研究に協力していただく患者さんに不利益が生じることはないと考えています。しかし、そうであっても臨床情報を本研究のために使用されたくない方は、ご連絡いただければ解析対象から除外します。

問い合わせ先：研究責任者： 恒光 健史

兵庫県立尼崎総合医療センター　救急集中治療科

〒660-8550 兵庫県尼崎市東難波町2-17-77

TEL： 06-6480-7000　FAX： 06-6480-7001
