# Supplementary material for: Effect of enterally administered sleep-promoting medication on the intravenous sedative dose and its safety and cost profile in mechanically ventilated patients: A retrospective cohort study
Source: PLoS One. 2021 Dec 20;16(12):e0261305. doi: 10.1371/journal.pone.0261305 (PMC8687529; doi:10.1371/journal.pone.0261305)
Supplement: S7 File — (DOCX) [file pone.0261305.s007.docx]

Title

Effect of enterally administered sleep-promoting medication on the intravenous sedative dose and its safety and cost profile in mechanically ventilated patients: a retrospective cohort study

Department of Critical care and emergency medicine

Takefumi Tsunemitsu

1. Purpose of the Study
2. Background and Significance of the Study
3. Research Subjects and Eligibility Criteria
4. Research Methods
5. Statistical Matters
6. Schedule or Duration of Study
7. Burden on Research Subjects and Anticipated Risks and Benefits
8. Modification, discontinuation, or termination of the research
9. Procedures for Obtaining Informed Consent from Research Subjects
10. Handling of Personal Information, etc.
11. Method of Storage and Disposal of Information
12. Content and Method of Reporting to the Director
13. Cost Burden and Gratuity for Research Subjects
14. Publication of Research Results
15. Research Funding and Conflict of Interest
16. Response to Consultation, etc., from Research Subjects, etc., and Related Persons
17. Outsourcing of Research Work
18. Research Implementation System
19. Reference Materials and Bibliography
20. Purpose of the Study

To study the relationship between changes in enteral doses of sleep-promoting medication and intravenous doses of sedatives and prognosis in patients intubated and undergoing ventilatory management.

1. Background and Significance of the Study

Sleep disruption, which is often observed in critically ill patients admitted to the ICU [1–3], has negative effects on patients and can cause a worse prognosis. It causes abnormalities in immune and metabolic endocrine functions, which play crucial roles in critically ill patients [4,5]. Additionally, it may be associated with the occurrence of delirium [6], noninvasive ventilation failure [7], and increased mortality [8]. Consequently, recent clinical guidelines have indicated the need for research methods to improve sleep [9].

Intravenous sedatives, including propofol and benzodiazepines, is administered to improve sleep efficiency [10]; however, they can negatively affect critically ill patients. Intravenous sedatives affect respiratory function, which causes apnea and hypoxia, and circulation, which causes bradycardia and hypotension [11–13]. Additionally, they may be associated with prolonged mechanical ventilation [14]. Moreover, intravenous sedatives tend to cause deep sedation [15], which is associated with a worse prognosis [16,17].

Sleep-promoting medication (SPM), including melatonin, ramelteon, and atypical antipsychotics, may improve sleep efficiency in critically ill patients [9]. A pilot randomized controlled trial reported that melatonin may improve nocturnal sleep efficiency [18]. Regarding safety, several studies have reported safe enteral administration of neuroactive medications [19,20]. Enteral SPM administration may be safely feasible for critically ill patients.

Currently, there have been no studies on the relationship between enteral SPM administration and the amount of intravenous sedatives, and the usefulness and safety of enteral SPM administration. We hypothesized that enteral SPM administration promotes sleep and reduces the intravenous sedative dose administered for sleep. This exploratory study aims to investigate the relationship between enteral SPM administration and the intravenous sedative dose. Further, we aim to examine the safety and cost of enteral SPM administration. We believe that this study is important in order to understand the effect of enteral SPM administration on mechanical ventilated patients.

1. Research Subjects and Eligibility Criteria

This study is conducted in the emergency intensive care unit at a 700-bedded tertiary medical care center in Japan between July 2015 and January 2020. We chose to start the study on July 2015 given that this was when our hospital opened.

The inclusion criteria are as followed: aged ≥ 15 years, having started mechanical ventilation within 24 hours of ICU admission, and requiring mechanical ventilation using an oral endotracheal tube for at least 48 hours. The exclusion criteria are as following: central nervous system disease (stroke, epilepsy, meningitis, encephalitis, etc.), cardiopulmonary arrest, traumatic brain injury, overdose, gastrointestinal tract impracticability (administration of continuous neuromuscular blockade, abdominal surgery, ileus, gastrointestinal bleeding, etc.), previous psychiatric or cognitive pathology, Child C hepatopathy, pregnancy, previous tracheostomy, ICU readmission, lacking body weight records, death in ICU, and not receiving propofol.

1. Research Methods

This single-center retrospective cohort study employs medical record information.

Exposure is enteral SPM administration during mechanical ventilation through an oral endotracheal tube. This study defines the following medications as SPM: trazodone, mianserin, quetiapine, and suvorexant [21–23]. Exposure is considered as present if these drugs were administered after enteral nutrition in the evening or before sleep. We divide patients into the following three groups: "administration within 48 hours of ICU admission (early administration)”, "administration after 48 hours of ICU admission (late administration)", and "no administration)". We set the cutoff at 48 hours since early sleep management appears to be more beneficial with consideration of sleep disruption pathophysiology [24] and enteral medication can be administered at the start of enteral feeding, which is recommended as within 48 hours of ICU admission [25,26]. This study does not consider ramelteon and benzodiazepines as exposures. This is because short-term ramelteon administration shows significant improvements in sleep; however, the differences were small [27]. Moreover, melatonin, which is a drug similar to ramelteon, has insufficient evidence regarding its improvement of sleep quality [28] and is not strongly recommended by the European guidelines [22]. Use of benzodiazepines in the ICU has a high incidence of adverse events [29]. Other antipsychotics (haloperidol, risperidone, etc.) are excluded from exposure since they are primarily given for agitation and are rarely used for sleep improvement in our ICU.

The primary outcome is the average daily propofol dose per body weight administered as a continuous sedative during mechanical ventilation through an oral endotracheal tube. The numerator is the total propofol dose administered as a continuous sedative during mechanical ventilation. On the other hand, the denominator is the body weight and number of days of mechanical ventilation. Propofol is chosen since it is the most commonly used sedative in our ICU and its increasing worldwide usage [30]. A continuous sedative is defined as one administered for > 1 hour. The physician on duty sets the target Richmond Agitation-Sedation Scale (RASS) for the sedative medication dose, with the nurses mainly taking initiative to increase or decrease it.

Other outcomes include the duration of mechanical ventilation using an oral endotracheal tube, length of ICU stay, delirium diagnosed based on the Confusion Assessment Method for ICU or Intensive Care Delirium Screening Checklist, and agitation defined as an RASS ≧ two points during mechanical ventilation with oral endotracheal tube. We collect data regarding adverse events, including self-extubation, liver damage requiring drug change, unplanned sedative administration (haloperidol, hydroxyzine, and benzodiazepines), and ventricular arrhythmia. The cost is calculated by totaling the amount of each enteral and intravenous drug extracted in this study during the mechanical ventilation through an oral endotracheal tube based on the drug price in June 2020. The conversion from yen to dollar was calculated at 108 yen per dollar, which is the rate as of June 2020. Additionally, we collect data regarding the administration of analgesics (acetaminophen and fentanyl), other intravenous sedatives (midazolam and dexmedetomidine), and other neuroactive enteral drugs (ramelteon, yokukansan, perospirone, risperidone, and benzodiazepines).

1. Statistical Matters

Continuous data are described using medians with interquartile range while categorical data using frequencies and percentages. We construct multiple linear regression models to examine the association between enteral SPM administration and the average daily propofol dose per body weight. We set the following variables as covariates for the primary study outcome: age, sex, pre-admission use of sleeping pills, diagnosis on ICU admission, maximum sequential organ failure assessment (SOFA) scores for one week after admission, intravenous dexmedetomidine administration, intravenous midazolam administration, enteral ramelteon administration, enteral benzodiazepine administration, average daily fentanyl dose per body weight, and acetaminophen administration. For analysis of other outcomes, logistic regression analysis is used for binary variables while multiple linear regression models were used for continuous variables. We set the following variables as covariates for secondary study outcomes: age, sex, Charlson comorbidity index, SOFA scores for one week after admission, diagnosis on ICU admission, and intravenous midazolam administration. All tests are two-sided, with a significance level of 0.05. Analyses are performed using EZR (version：1.36) [31].

1. Schedule or Duration of Study

Date of approval by the Clinical Research Review Committee - December 2022

(Study period: July 2015 to January 2020)

1. Burden on Research Subjects and Anticipated Risks and Benefits

There are no burdens or risks to the research subjects by participating in this research.

No direct benefit to the individual research subject will result from participation in this study. The results of the study may be beneficial to future advances in good sedation practices for patients requiring ventilator management.

1. Modification, discontinuation, or termination of the research

Any changes or revisions to the research protocol must be approved in advance by the Ethics Committee.

If the ethics committee recommends or directs that the research be stopped, the principal investigator shall stop the research. When the decision to discontinue or suspend the research is made, the researcher shall promptly report to the Director in writing with the reasons for the decision.

At the end of the research, the principal investigator shall promptly submit a research termination report to the director of the hospital.

1. Procedures for Obtaining Informed Consent from Research Subjects

No written or verbal consent will be obtained from the research subjects, as this research will not involve the acquisition of new samples or information, but will be conducted using only existing information. Information about the research will be disclosed to the research subjects (posted in the hospital), and the research subjects will be guaranteed the opportunity to refuse the research being conducted.

1. Handling of Personal Information, etc.

Those involved in the research shall comply with the "Ethical Guidelines for Medical Research Involving Human Subjects," the "Act on the Protection of Personal Information," and applicable laws, regulations, and ordinances regarding the handling of personal information. When handling information obtained through research, sufficient consideration will be given to the protection of the confidentiality of research subjects, and codes or numbers will be assigned to research subjects so that specific individuals cannot be identified. Correspondence tables will be stored on the electronic medical record network by the principal investigator, and information that can identify individuals will not be taken outside the network. For analysis, only anonymized processed information will be analyzed using the individual's computer. In the event of refusal, the principal investigator will delete the information from the anonymized processed information. When the results of this research are published, they will not contain information that can identify individual research subjects. In addition, the information obtained in this research will not be used for any purpose other than the purpose of this research.

1. Method of Storage and Disposal of Information

Principal investigators shall store documents related to the conduct of research, etc. (e.g., copies of application documents, notification documents, lists of identification codes for research subjects, consent forms, case reports, and other documents or records necessary to assure the reliability of data) in a lockable locker in the medical office. The storage period shall be until the later of five years after the date of the report on the completion of the research or three years after the date of the report on the final publication of the research results. At the end of the storage period, paper media will be shredded and destroyed. Other media will be destroyed by an appropriate method.

1. Content and Method of Reporting to the Director

The following cases shall be reported to the Director in writing.

1) When facts or information that impairs or may impair the ethical validity or scientific rationality of the research, and that may affect the continuation of the research, are obtained.

2) When facts or information that impair or may impair the appropriateness of the conduct of the research or the reliability of the research results are obtained.

3) When the research is terminated (discontinued)

4) Progress of the research (once a year)

1. Cost Burden and Gratuity for Research Subjects

There will be no cost to the research subjects for participating in the research. Also, no honorarium will be given.

1. Publication of Research Results

Make presentations at international and domestic conferences and papers. At the time of publication, sufficient care will be taken not to use any information that identifies individuals.

1. Research Funding and Conflict of Interest

This research will be conducted with research funds from the department to which the principal investigator belongs. In addition, the researcher of this research shall declare the necessary matters to the Clinical Research Conflict of Interest Committee in accordance with the "Management Rules for Conflict of Interest of Amagasaki General Medical Center" and obtain its review and approval.

1. Response to Consultation, etc., from Research Subjects, etc., and Related Persons

The point of contact for consultation from research subjects and their related persons shall be the principal investigator, and the contact information shall be included in the public document.

1. Outsourcing of Research Work

There is no outsourcing of research-related tasks.

1. Research Implementation System

Department of Emergency and Critical Care Medicine, Hyogo Prefectural Amagasaki General Medical Center

Principal Investigator: Takefumi Tsunemitsu, Department of Emergency Medicine

1. Reference Materials and Bibliography
2. Cooper AB, Thornley KS, Bryan Young G, et al. Sleep in Critically Ill Patients Requiring Mechanical Ventilation. Chest. 2000;117(3):809-18.
3. Freedman NS, Gazendam J, Levan L, et al. Abnormal Sleep/Wake Cycles and the Effect of Environmental Noise on Sleep Disruption in the Intensive Care Unit. Am J Respir Crit Care Med. 2001;163(2):451-7.
4. Elliott R, McKinley S, Cistulli P, et al. Characterisation of sleep in intensive care using 24-hour polysomnography: An observational study. Crit Care. 2013;17(2):R46.
5. Spiegel K, Leproult R, Cauter E Van. Impact of sleep debt on metabolic and endocrine function. Lancet. 1999;354(9188):1435-9.
6. Spiegel K, Sheridan JF, Van Cauter E. Effect of sleep deprivation on response to immunization. JAMA. 2002;288:1471–2.
7. Fadayomi AB, Ibala R, Bilotta F, et al. A Systematic Review and Meta-Analysis Examining the Impact of Sleep Disturbance on Postoperative Delirium. Crit Care Med. 2018;46(12):e1204-e1212.
8. Campo FR, Drouot X, Thille AW, et al. Poor sleep quality is associated with late noninvasive ventilation failure in patients with acute hypercapnic respiratory failure. Crit Care Med. 2010;38(2):477-85.
9. Boyko Y, Toft P, Ørding H, et al. Atypical sleep in critically ill patients on mechanical ventilation is associated with increased mortality. Sleep Breath. 2019;23:379–88.
10. Devlin JW, Skrobik Y, Gélinas C, et al. Clinical Practice Guidelines for the Prevention and Management of Pain, Agitation/Sedation, Delirium, Immobility, and Sleep Disruption in Adult Patients in the ICU. Crit Care Med. 2018;46(9):e825-e873.
11. Brito RA, do Nascimento Rebouças Viana SM, Beltrão BA, et al. Pharmacological and non-pharmacological interventions to promote sleep in intensive care units: a critical review. Sleep Breath. 2020;24(1):25-35.
12. McCollum JS, Dundee JW, Halliday NJ, et al. Dose response studies with propofol ('Diprivan’) in unpremedicated patients. Postgrad Med J. 1985;61 Suppl 3:85-7.
13. Xia ZQ, Chen SQ, Yao X, et al. Clinical benefits of dexmedetomidine versus propofol in adult intensive care unit patients: A meta-analysis of randomized clinical trials. J Surg Res. 2013;185(2):833-43.
14. Chawla N, Boateng A, Deshpande R. Procedural sedation in the ICU and emergency department. Curr Opin Anaesthesiol. 2017;30(4):507-512.
15. Kollef MH, Levy NT, Ahrens TS, et al. The use of continuous IV sedation is associated with prolongation of mechanical ventilation. Chest. 1998;114:541–8.
16. Jackson DL, Proudfoot CW, Cann KF, et al. The incidence of sub-optimal sedation in the ICU: A systematic review. Crit Care. 2009;13(6):R204.
17. Shehabi Y, Bellomo R, Kadiman S, et al. Sedation intensity in the first 48 hours of mechanical ventilation and 180-day mortality: A multinational prospective longitudinal cohort study. Crit Care Med. 2018;46(6):850-859.
18. Tanaka LMS, Azevedo LCP, Park M, Schettino G, et al. Early sedation and clinical outcomes of mechanically ventilated patients: A prospective multicenter cohort study. Crit Care. 2014;18(4):R156.
19. Bourne RS, Mills GH, Minelli C. Melatonin therapy to improve nocturnal sleep in critically ill patients: Encouraging results from a small randomised controlled trial. Crit Care. 2008;12(2):R52.
20. Dube KM, DeGrado J, Hohlfelder B, et al. Evaluation of the Effects of Quetiapine on QTc Prolongation in Critically Ill Patients. J Pharm Pract. 2018;31(3):292-297.
21. Hatta K, Kishi Y, Wada K, et al. Preventive effects of suvorexant on delirium: A randomized placebo-controlled trial. J Clin Psychiatry. 2017;78(8):e970-e979.
22. Yi X yan, Ni S fen, Ghadami MR, et al. Trazodone for the treatment of insomnia: a meta-analysis of randomized placebo-controlled trials. Sleep Med. 2018;45:25-32.
23. Riemann D, Baglioni C, Bassetti C, et al. European guideline for the diagnosis and treatment of insomnia. J Sleep Res. 2017;26:675–700.
24. Herring WJ, Connor KM, Snyder E, et al. Effects of suvorexant on the Insomnia Severity Index in patients with insomnia: analysis of pooled phase 3 data. Sleep Med. 2019;56:219-223.
25. Pisani MA, D’Ambrosio C. Sleep and Delirium in Critically Ill Adults: A Contemporary Review. Chest. 2020;157(4):977-984.
26. McClave SA, Taylor BE, Martindale RG, et al. Guidelines for the Provision and Assessment of Nutrition Support Therapy in the Adult Critically Ill Patient: Society of Critical Care Medicine (SCCM) and American Society for Parenteral and Enteral Nutrition (A.S.P.E.N.). J Parenter Enter Nutr. 2016;40:159–211.
27. Singer P, Blaser AR, Berger MM, et al. ESPEN guideline on clinical nutrition in the intensive care unit. Clin Nutr. 2019;38(1):48-79.
28. Kuriyama A, Honda M, Hayashino Y. Ramelteon for the treatment of insomnia in adults: A systematic review and meta-analysis. Sleep Med. 2014;15(4):385-92.
29. Lewis SR, Pritchard MW, Schofield-Robinson OJ, et al. Melatonin for the promotion of sleep in adults in the intensive care unit. Cochrane Database Syst Rev. 2018;5(5):CD012455.
30. Kok L, Slooter AJ, Hillegers MH, et al. Benzodiazepine use and neuropsychiatric outcomes in the ICU: A systematic review. Crit. Care Med. 2018;46(10):1673-1680.
31. Owen GD, Stollings JL, Rakhit S, et al. International analgesia, sedation, and delirium practices: A prospective cohort study. J Intensive Care. 2019;7:25.
32. Kanda Y. Investigation of the freely available easy-to-use software “EZR” for medical statistics. Bone Marrow Transplant. 2013;48:452–8.
