# Supplementary material for: Effect of enterally administered sleep-promoting medication on the intravenous sedative dose and its safety and cost profile in mechanically ventilated patients: A retrospective cohort study
Source: PLoS One. 2021 Dec 20;16(12):e0261305. doi: 10.1371/journal.pone.0261305 (PMC8687529; doi:10.1371/journal.pone.0261305)
Supplement: Attachment — Submitted filename: Response to Reviewers.docx [file pone.0261305.s009.docx]

Emily Chenette

Editor-in-Chief

*PLOS ONE*

Dear Editor,

Manuscript No: PONE-D-21-05291

Title: Effect of enterally administered sleep-promoting medication on the intravenous sedative dose and its safety and cost profile in mechanically ventilated patients: A retrospective cohort study

We would like to express our sincere thanks to you and the reviewers for the thorough review of our manuscript and for the opportunity to submit a revised and improved version. We have carefully reviewed the comments and revised the manuscript on the basis of the reviewers’ comments. Our point-by-point responses to the reviewers’ comments are listed below.

Journal Requirements:

●Please review your reference list to ensure that it is complete and correct. If you have cited papers that have been retracted, please include the rationale for doing so in the manuscript text, or remove these references and replace them with relevant current references. Any changes to the reference list should be mentioned in the rebuttal letter that accompanies your revised manuscript. If you need to cite a retracted article, indicate the article’s retracted status in the References list and also include a citation and full reference for the retraction notice.

Response: I have checked the references and did not make any changes.

Reviewer #1:

●Thank you for the opportunity to review this revised manuscript. I would like to thank the authors for addressing my concerns. No difference in primary and secondary outcomes between the groups when considered EA and LA together were reported. This was adequately commented on. I am still not sure about the adequacy of the statistical analysis. However, the authors stated they consulted a Statistician, who they should consider to acknowledge in the appropriate section.

Response: We agree with the reviewer’s assessment. Dr. Yuki Kataoka is an expert in statistics, and He performed the statistical processing for this study. In the Authors' contributions section, We’ve already described his contribution to the statistical process.

●Please consider modifying the sentence “The SOFA score and Charlson comorbidity index were similar in each group”, since no statistical test for assessing among-group difference was performed, similar to the sentence regarding age, gender and BMI.

Response: We thank the reviewer for providing these insights. We have rewritten the sentences according to the reviewer’s comments: “The SOFA score and Charlson comorbidity index were similar in each group” to “The SOFA score and Charlson comorbidity index were 6 and 0 in the EA group, 6 and 0 in the LA group, and 7 and 0 in the NA group.”

Again, thank you for giving us the opportunity to strengthen our manuscript with your valuable comments and queries. We have worked hard to incorporate your feedback and hope that these revisions persuade you to accept our submission.

Sincerely,

Takefumi Tsunemitsu

Department of Emergency and Critical Care Medicine, Hyogo Prefectural Amagasaki General Medical Center 2-17-77 Higashinaniwa-cho, Amagasaki, Hyogo, 660-8550, Japan

Phone: 81-6-6480-7000

Fax: 81-6-6480-7001

E-mail: tsunemitsu0730@yahoo.co.jp
